# Subgraph extraction and graph representation learning for single cell Hi-C imputation and clustering

**DOI:** 10.1093/bib/bbad379

**Published:** 2023-12-01

**Authors:** Jiahao Zheng, Yuedong Yang, Zhiming Dai

**Affiliations:** School of Computer Science and Engineering, Sun Yat-Sen University, 510006 Guangzhou, China; School of Computer Science and Engineering, Sun Yat-Sen University, 510006 Guangzhou, China; School of Computer Science and Engineering, Sun Yat-Sen University, 510006 Guangzhou, China

**Keywords:** single cell, Hi-C, 3D genome, graph neural network

## Abstract

Single-cell Hi-C (scHi-C) technology enables the investigation of 3D chromatin structure variability across individual cells. However, the analysis of scHi-C data is challenged by a large number of missing values. Here, we present a scHi-C data imputation model HiC-SGL, based on Subgraph extraction and graph representation learning. HiC-SGL can also learn informative low-dimensional embeddings of cells. We demonstrate that our method surpasses existing methods in terms of imputation accuracy and clustering performance by various metrics.

## INTRODUCTION

Chromatin conformation capture technologies such as Hi-C [1] can generate a genome-wide interaction map, which indicates the frequency of contact between any two loci on the chromosome. These data reveal the spatial organization of the genome at multiple scales, including nuclear compartmentalization [1–4], chromosomal territories [5–7], topologically associating domains (TADs) [8] and chromatin loops [2]. The spatial architecture of the genome is related to biological processes such as DNA replication and transcription [2, 9–11]. The recent advances in single-cell Hi-C (scHi-C) technology [12–18] enable the analysis of genome structure at the single-cell level. Unlike bulk Hi-C, which represents the average contact map of millions of cells, scHi-C can capture the heterogeneity and similarity among cells, and thus better elucidate the link between cell-specific structure and function, and facilitate identification of cell types.

However, the first challenge for the computational analysis of scHi-C data is data sparsity. Data sparsity refers to a matrix of numbers that includes many zeros or values. The effective part (non-zero value) of the scHi-C contact matrix constitutes less than 1% of the entire matrix. One cause of data sparsity is a lot of missing values due to technological limit. Missing value in the contact matrix does not necessarily indicate the absence of interaction between the corresponding loci; instead, it might indicate that the interaction is undetected by scHi-C. Recovering missing data from existing data to enhance data quality is important for downstream tasks such as reconstructing 3D structures from contact maps [19]. Therefore, the accurate imputation of scHi-C data is very necessary, and a good imputation method can facilitate the subsequent analysis of scHi-C data.

To the best of our knowledge, three imputation methods for scHi-C data have been proposed, such as scHiCluster [20], which combines convolution and random walk with restart; Higashi [21, 22], which employs a hypergraph learning model; scDEC-Hi-C [23], which introduces a convolutional generative neural network model. It is reasonable for the use of graphs to model scHi-C data because the contact map is essentially an adjacency matrix that reflects the genomic structure network. We represent the scHi-C dataset as a graphset: $\{G_i = (V_i, E_i)\}$, where *i* represents the *i*th cell, each vertex corresponds to a chromosomal segment(bin), and each edge $e(u, v)(\in E_i) = c$ denotes the number of interactions between bin $u$ and bin $v$. This transforms imputation into a link prediction task for all scHi-C graphs.

**Figure 1:**
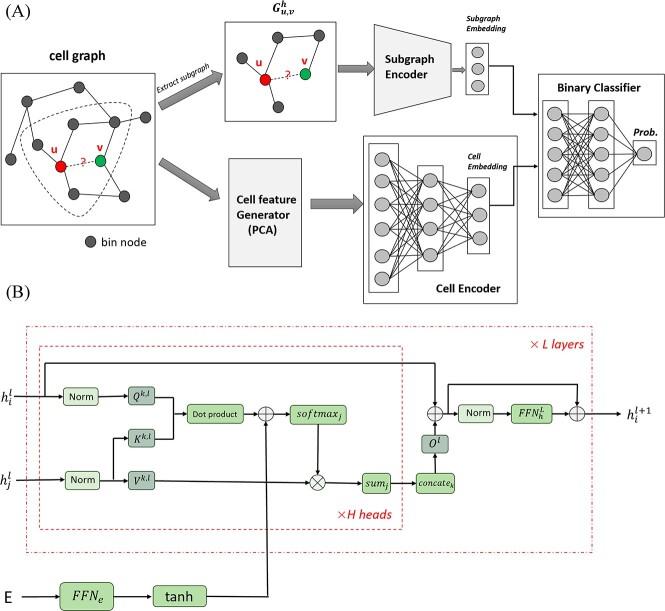
(**A**) The structure of the **HiC-SGL** model. It consists of an encoder and a decoder. The encoder extracts the local feature of each edge and the global feature of the cell graph. The decoder estimates the likelihood of each edge being present in the cell map using the encoded feature. (**B**) Subgraph Encoder, implemented by variant of Transformer on graph neural networks. $h_i^l, h_j^l$: Node Embeddings of layer $l$; E: adjacency matrix of subgraph.

The existence of links depends on the intra-graph structure and the inter-graph relationships. Intuitively, the connection of two nodes on the scHi-C graph reflects their spatial proximity. Based on this, we can infer that if two nodes share many common neighbors or if the nodes of adjacent chromosome segments upstream and downstream of one node are connected to the other node, then the distance of these two nodes tends to be small, thus their probability of connection will be high. This predefined graph structural feature computed based on the link context is called a heuristic. By defining a suitable heuristic function, it has achieved great success in various link prediction tasks [24–29]. The random walk with restart used in scHiClutser is a heuristic method to compute the transition probability between two nodes as a measure of their closeness. Instead of manually searching for suitable heuristics, in our work, heuristic information helpful for link prediction is automatically computed by extracting k-hop neighborhood subgraphs of the links and fed into a subgraph encoder. Similar strategies have has also been adopted in many previous studies with great success [30–32]. In addition, we compute an embedding that represents the global information of the cell and share it among the links of the corresponding cells, not only as a complement to the local information, but also as a way to capture the differences in density, type, cycle state and connection rules among cells. We use these two kinds of information together to make predictions.

In addition, we adapt the transformer architecture [33] to graph neural networks to implement the subgraph encoder. This is inspired by previous works that modify the attention mechanism and other components of the transformer to encode the structural information of the graph [34–36]. Unlike conventional graph neural networks such as graph attention network [37–40], these methods do not restrict information propagation to neighboring nodes, but adopt a fully connected graph strategy, which can enhance their expressive power, but increases their space-time complexity. In our model, we apply the encoder only to subgraphs that are much smaller than the original graph, so this drawback of space-time complexity can be mitigated.

In conclusion, we present HiC-SGL, a novel end-to-end model for imputing scHi-C data based on subgraph extraction and graph representation learning. Our model effectively integrates the intra-cellular link structure and the inter-cellular variability as two main sources of information for imputation, and produces low-dimensional cell embeddings that capture cell types and states. We demonstrate that our model outperforms existing methods in terms of imputation accuracy and clustering performance by various metrics.

**Figure 2:**
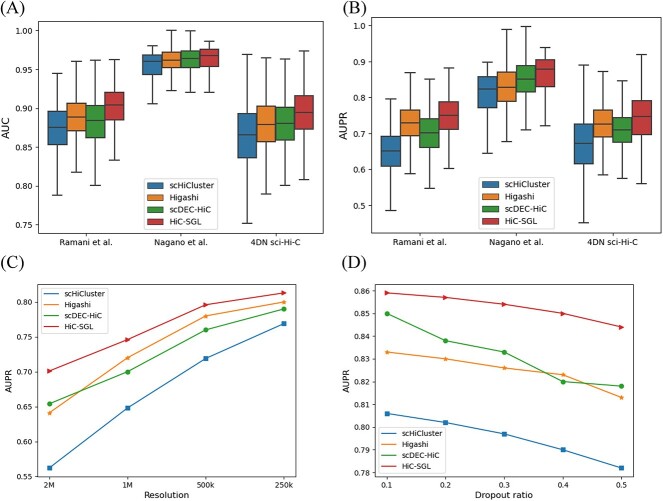
(**A**) Imputation accuracy for all cells on the three datasets (AUC), (**B**) imputation accuracy for all cells on the three datasets (AUPR), (**C**) imputation accuracy at different resolution on Remani et al. dataset (AUPR). (**D**) imputation accuracy at different dropout ratio on Nagano et al. dataset (AUPR).

## RESULTS

### Overview of HiC-SGL

The model has an encoder-decoder structure (Figure 1A). The encoder captures both the local feature of each edge (i.e. subgraph embedding) and the global feature of the cell graph (i.e. cell embedding). The decoder predicts the probability of each edge’s existence in the cell graph based on the encoded feature. The encoder has two branches: a cell-encoder and a subgraph-encoder. The cell-encoder applies dimensionality reduction methods such as PCA to obtain the initial cell features from the cell contact matrix, and then learns the latent representation of each cell using a two-layer MLP. The subgraph-encoder extracts a k-hop neighborhood subgraph around the edge $(u, v)$ to be imputed in the cell graph $G_i = (V_i,E_i)$, and feeds it into a subgraph encoder to obtain the latent representation of the subgraph. We propose a method for subgraph encoding using a variant of the transformer architecture in the context of graph neural networks (Figure 1B). Specifically, we leverage the adjacency matrix of the subgraph to compute the weights between links, which are then utilized as bias terms to modify the attention weights. This enables the integration of subgraph structure information into the node update process.

Our model leverages the structural information within the cell by extracting and encoding subgraphs of k-hop neighbors. Since scHi-C data exhibits strong locality, we assume that most of the information for imputation is contained in the neighborhood subgraphs. By extracting and encoding these subgraphs, we avoid computing on the whole graph and reduce the time and space complexity while preserving most of the information. The cell encoding provides global information that complements the local information and exploits the variability between cells for imputation. If $cell_i$ and $cell_j$ have very similar positive and negative sample sets, they will have similar cell embeddings. Furthermore, when a subgraph of the link to be predicted in $cell_j$ happens to be a positive sample in $cell_i$, then $cell_j$ will tend to predict it as a positive sample.

### HiC-SGL can accurately impute scHi-C contact maps

In this part, we demonstrate whether HiC-SGL can more accurately impute scHi-C data compared with existing methods through experiments on public datasets. We evaluate our model on three datasets: Ramani et al. dataset [14], Nagano et al. dataset [12] and 4DN sci-Hi-C dataset [41]. We generate a simulated dataset by downsampling these datasets for link prediction testing (see methods for details of these datasets and data processing procedure). We compare our method with existing scHi-C data imputation methods: scHiCluster [20], Higashi [21] and scDEC-Hi-C [23], using AUPR and AUC metrics to assess the imputation results. These quantitative results (Figure 2A and B) show that our method achieves significantly higher imputation accuracy than the other three methods on the three datasets and results in Supplementary Figure 1 show imputation performance for individual cell types in these datasets. To ensure the robustness of the experiment, we also test the imputation performance of our model at different resolutions and dropout ratios. The results indicate that our model surpasses other methods at different resolutions (Figure 2C) and that its prediction accuracy declines more slowly than other methods as the dropout ratio increases (Figure 2D). We downsample these datasets to the same number of cells and the same number of contacts per cell at the same resolution and HiC-SGL still outperforms other methods under this condition (Supplementary Figure 2).

### Ablation study shows the importance of cell-encoder and subgraph-encoder

We conduct a comparative analysis of three models derived from our original model by modifying its components as follows:

- **Cell-Encoder-only:** Eliminate the subgraph encoder and replaces the subgraph embedding with the concatenation of the initial embeddings of the source and target nodes of the edge to be predicted.
- **Sub-Encoder-only:** Eliminate the cell encoder and feeds only the subgraph embedding to the decoder.
- **HiC-SGL-GAT:** Replace Transformer-based subgraph encoder in our model by GAT.

The results (Table 1) show that these three models perform significantly worse than the original model, indicating that both the subgraph encoder and the cell encoder are essential. This implies that scHi-C data imputation should leverage both the spatial structural features within cells and the intercellular similarities and differences, and the model utilizing transformer variant of GNNs outperforms the model using conventional Graph Neural Networks (GNNs) due to the enhanced expressiveness.

**Table 1:** Ablation study on three datasets (AUPR)

	Ramani et al.	Nagano et al.	4DN sci-Hi-C
Cell-Encoder-only	0.725	0.792	0.718
Sub-Encoder-only	0.732	0.842	0.672
HiC-SGL-GAT	0.741	0.844	0.736
**HiC-SGL**	**0.747**	**0.859**	**0.742**

Using the subgraph encoder with the transformer variant of GNNs is shown to be superior to traditional GNNs alternatives such as GCN or GAT. Furthermore, upon removing attention bias, our method exhibits a significant decrease in imputation performance across three different datasets (Supplementary Table 1). This underscores the effective utilization of graph structure information by our model in the context of link prediction. We provide insight into our trained model by calculating attention weights and attention biases on certain subgraphs (Supplementary Figures 3 and 4). Notably, we observe discrepancies between attention biases and the adjacency matrices of subgraphs, as well as variations in attention weight matrices across different layers and heads. We posit that the advantage of the transformer variant of GNNs over GCN or GAT lies in its ability to offer multiple message-passing mechanisms between vertices in the subgraph, extending beyond the transmission of messages’ limited to existing links within the subgraph. This feature enhances the expressive power of the model across different layers and heads.

We compare the performance of our model with varying neighborhood subgraph sampling hop counts, *k* = 1,2,3, on three datasets (Supplementary Table 2). We observed that the model performs sufficiently well with *k* = 1: it achieved optimal performance at *k* = 1 on the Remani et al. and Nagano et al. datasets, while on the 4DN sci-Hi-C dataset, the model reached its peak performance at *k* = 2.

### HiC-SGL embeddings reflect cell types and states

**Figure 3:**
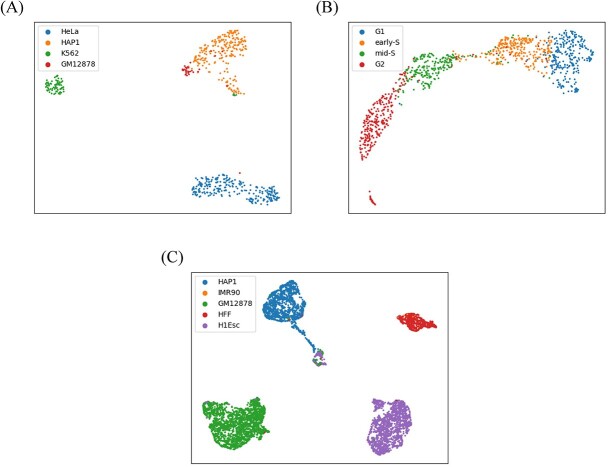
UMAP visualization of HiC-SGL cell embedding on three datasets. (**A**) Ramani et al. dataset. (**B**) Nagano et al. dataset. (**C**) 4DN sci-Hi-C dataset.

We evaluate the clustering performance of our method on three datasets mentioned above, which aim to identify distinct cell types in heterogeneous cell populations using scHi-C data. We visualize the learned cell embeddings in a two-dimensional space by applying the uniform manifold approximation and projection (UMAP) method(Figure 3A–C). For Ramani et al. dataset [14] and 4DN-sci-Hi-C dataset [41] with discrete cell states, we use K-means clustering and compare the predicted labels with the true cell types using the Adjusted Rand Index (ARI) metric. For the Nagano et al [12]. dataset with continuous cell states, we use the Average Circular ROC (ACROC) [42] metric. The results (Table 2) suggest that our method achieves comparable performance with existing methods on the Ramani et al. dataset and outperforms them on 4DN-sci-Hi-C dataset and Nagano et al. dataset.

### HiC-SGL can help identify the A/B compartments

**Figure 4:**
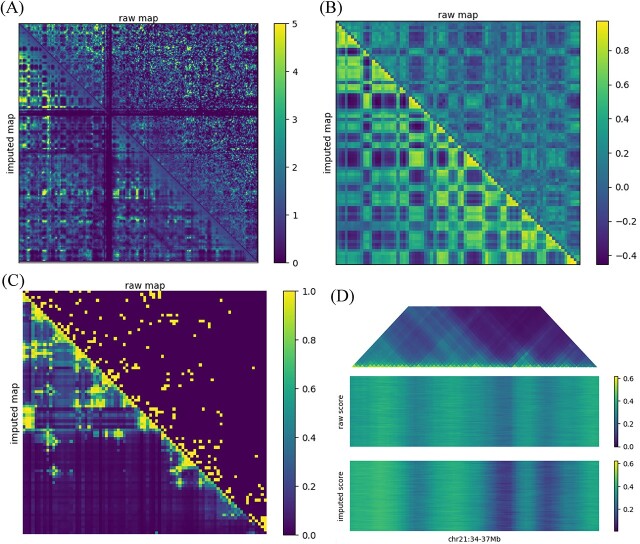
(**A**) Distance normalized Hi-C map(chr2) of Ramani et al. datasets before and after HiC-SGL imputation. (**B**) Pearson correlation Hi-C map(chr2 0-80M) of Ramani et al. datasets before and after HiC-SGL imputation. (**C**) A single-cell contact map derived from STORM 3D genome imaging data before and after HiC-SGL imputation. (**D**) Insulation scores of the STORM 3D genome imaging dataset before and after imputation, with each row corresponding to the insulation scores of a specific cell. The top represents the merged imputed scHi-C map.

Lieberman-Aiden et al. [1] discovered that each chromosome can be partitioned into two groups of loci (designated as A and B), where interactions within each group are enhanced, while interactions between the groups are diminished. Following the method in their work, we merge the contact matrices of single-cell Hi-C data on Ramani et al. datasets to obtain pseudo-bulk Hi-C data M. Subsequently, each entry in M is divided by the genome-wide average contact probability at that genomic distance, resulting in a normalized contact matrix M*. Finally, we compute the Pearson correlation matrix C for M*, where cij represents the Pearson correlation between the ith row and jth column of M*. We perform this process separately for single-cell Hi-C contact matrices before and after imputation. It can be observed that, compared with the raw data, the imputed Pearson correlation matrix exhibits a more distinct checkerboard pattern (Figure 4B). This indicates that HiC-SGL is capable of assisting in the identification of A/B compartments of chromosomes.

### HiC-SGL reveals clearer single-cell TAD boundaries

We utilize scHi-C data simulated from multiplexed STORM 3D genome imaging data [43], covering a 2.5-Mb region on chr21 derived from 11,631 cells at a 30-Kb resolution. By calculating insulation scores for each cell, we obtain insulation score matrices for the scHi-C data before and after imputation. We denote the contact map as M and the window size as w. The insulation score s at position x is the ratio of the total interactions between the windows of size w on either side of x to the total interactions within a window centered at x with a radius of w reflects the likelihood of x being a TAD boundary, with a smaller score indicating a higher probability. We observe that the cell interaction maps imputed by HiC-SGL exhibited clearer TAD structures (Figure 4C). Additionally, in the imputed insulation matrices, we identify three TAD boundaries (chr21:35.68 Mb, chr21:36.37 Mb and chr21:36.69 Mb) that were more clearer compared to the pre-imputation data (Figure 4D). As observed in previous studies, certain TAD boundaries have been found to be stable across different cell types [44], the results presented above suggest that within this single-cell dataset, there are stable boundaries that are shared by most cells.

**Table 2:** Clustering performace of cell embeddings on Ramani et al. dataset (ARI), Nagano et al. dataset (ACROC) and 4DN sci-Hi-C dataset (ARI)

	Ramani et al.	Nagano et al.	4DN sci-Hi-C
scHiCluster	0.794	0.759	0.733
Higashi	0.819	0.932	0.818
scDEC-Hi-C	**0.828**	0.925	0.832
**HiC-SGL**	0.821	**0.945**	**0.856**

## DISCUSSION

In this work, we propose HiC-SGL, a scHi-C imputation model based on subgraph extraction and graph representation learning, which can accurately impute scHi-C data and identify cell types. The core of the model is to encode local information from the subgraphs of the edges to be predicted, global information from the cell graph, and combine them for link prediction. The advantage of the model lies on its architecture, while the implementation of each component still has room for improvement, such as the extraction range of subgraphs, the acquisition of initial features of cells, the negative sampling method during training, etc. Furthermore, in contrast to bulk HiC data, the inherent sparsity of single-cell HiC data restricts its applicability for imputation or other calculations at a high resolution. Consequently, there remains a need for more work to facilitate the analysis of single-cell HiC data.

## METHOD

### ScHi-C data and data processing procedure

In this work, as in previous studies, we use three public datasets including Ramani et al. [14] (GEO: GSE84920), 4DN sci-Hi-C [41] (4dn Data portal: 4DNFIY7AMPUJ, 4DNESUE2NSGS, 4DNESIKGI39T, 4DNES1BK1RMQ and 4DNESTVIP977), Nagano et al. [12] (GEO: GSE94489) and multiplexed STORM 3D genome imaging data [43] (https://github.com/BogdanBintu/ChromatinImaging). The interaction pairs from the Nagano et al.and Ramani et al. datasets were downloaded from the corresponding GEO repository. For 4DN sci-Hi-C, we downloaded the FASTQ files and processed them with the recommended processing pipeline (https://github.com/VRam142/combinatorialHiC).

We preprocess each dataset by applying Binning process at a specific resolution, filtering out cells with too low or too high read counts, and organizing each dataset into a graphset. For the Ramani et al. dataset, which contains 621 cells of four human cell types (GM12878, HAP1, HeLa and K562) with a median read count of 7800, we use 1-Mb resolution and 2000–40 000 read count threshold. For the 4DN sci-Hi-C dataset, which contains 12 005 cells of five human cell types (GM12878, H1ESC, HAP1, HFFc6 and IMR90) with a median read count of 2500, we use 1-Mb resolution and 1000–2000 read count threshold. For the Nagano et al. dataset, which contains 1175 cells of four cell types (G1, mid-S, late-S, G2) with a median read count of 61 300, we use 250-kb resolution and 20 000–400 000 read count threshold.

We randomly remove 10% of the existing edges in each dataset and use them as positive samples for the test data. We then sample non-existing edges as negative samples, the number of which is four times more than that of positive samples, The remaining 90% of the existing edges and an five times number of non-existing edges are used to construct the training data.

In the case of multiplexed STORM 3D genome imaging data involving 11,631 imaged chromosome regions (chr21:34.6Mb-37.1Mb) as presented in [43], we initially convert the 3D coordinates of consecutive 30-Kb bins into a spatial distance map, resulting in a size of 83 $\times $ 83 for each cell. We employ the inverse spatial distance map as our reference data (referred to as the probability map) and randomly select 200 reads with replacement, proportionally to the value of each entry in the probability map.

### Model architecture and forward propagation

Our model has two parts: an encoder and a decoder (Figure 1A). The encoder learns both the local and global features of the cell graph from the edge subgraphs and the cell contact matrix. The decoder uses these features to predict the edges in the cell map. The encoder has two branches: one for cells and one for subgraphs. The cell encoder reduces the dimension of the cell contact matrix (by PCA) and then uses an MLP to get the cell features. The subgraph branch gets a h-hop subgraph around each edge and encodes it with a transformer-based model that uses the subgraph adjacency matrix to modify the attention weights (Figure 1B).

**Cell encoder.** For one scHi-C dataset, We represent it as $S = \{G_i = (V_i, E_i)\},\ |S | = m, \ |V_i| = n$. Flatten all adjacency matrix $A_i \in R^{n \times n}$ of $G_i$ to vector $A_i^{^{\prime}}\in R^{1 \times n^2}$, merge $[A_1^{^{\prime}}, A_2^{^{\prime}},...,A_m^{^{\prime}}]$ and apply PCA to get low dimension vector $[B_1, B_2,...,B_m]$. Input $B_i$ into two-layer MLP and we get cell embedding $C_i$: 


(1) \begin{align*}& C_i = W_2^c\max(0, W_1^c B_i + b_1^c) + b_2^c\end{align*}

**Subgraph encoder.**  $(u,v)$ is vertex tuple in $G_i$ to be predicted. First extact h-hop subgraph $G_{u,v}^h = (V^{^{\prime}}, E^{^{\prime}})$(induced from $G_i$ by the vertex subset $\lbrace i|d(i,u) <= h \ or \ d(i,v) <= h\rbrace $), $|V^{\prime}| = n^{\prime}$.

Initialize embeddings $\{x_1, x_2,..., x_{n}\}$ of the vertices representing the fragments of each genome, embeddings $\{z_1, z_2,..., z_{n^{\prime}}\}$ representing node centrality, both are initialized from standard normal distribution. Initial embedding of $v_i$ in $G_{u,v}^h$ is 


(2) \begin{align*}& h_i^0 = x_{i}+ z_{deg(v_i)}\end{align*}

$D \in R^{n^{\prime} \times n^{\prime}}$ is adjacency matrix of $G_{u,v}^h$. Input it to two-layer MLP and get *E* as bias of attention matrix: 


(3) \begin{align*} & E = Tanh(ReLU(DW_1^e + b_1^e)W_2^e + b_2^e) \end{align*}

(4) \begin{align*} & A_{ij}^l = \frac{(h_i^l W_Q^l )(h_j ^l W_K^l)^T} {\sqrt{d}} + E_{ij} \end{align*}

Based on the Multi-Head Attenti on(MHA) in the transformer [33], apply equations (3, 4) to modify the attention weight to get MHA*. Node embedding update process of each layer is described by the following equation: 


(5) \begin{align*} & H^0 = [h_0 ^ 0, h_1 ^0,..., h_{m} ^ 0] \end{align*}

(6) \begin{align*} & \hat{H}^{l} = MHA^*(LN(H^{l-1})) + H^{l-1} \end{align*}

(7) \begin{align*} & H^l = FFN(LN(\hat{H}^l)) + \hat{H}^{l} \end{align*}

**Decoder**. Implement by two-layer MLP, receives Embedding generated from encoder and outputs link probability: 


(8) \begin{align*}& \hat{y}_{i, u, v} = Sigmoid( ReLU((C_i|h_u^L|h_v^L)W_1^d + b_1^d)W_2^d + b_2^d)\end{align*}

### Training

**Loss function.** In HiC-SGL, the neural network generates a score, denoted as $\hat{y}_{i,u,v}$, for each triplet $(c_i, b_u, b_v)$. The objective of training the neural network is to minimize the disparity between the predicted score ($\hat{y}_{i,u,v}$) and the target score ($y_{i,u,v}$), which represents the likelihood of pairwise interaction between bin nodes $bu$ and $bv$ in cell $c_i$ as observed in the dataset. To achieve this, the model employs a binary classification loss (cross-entropy). In this loss function, triplets associated with non-zero entries in the single-cell contact maps are treated as positive samples, while the remaining triplets are considered negative samples. The binary classification loss is 


(9) \begin{align*}& Loss = -\sum_{i,u,v}y_{i,u,v}\log(\hat{y}_{i,u,v}) + (1-y_{i,u,v})\log(1 - \hat{y}_{i,u,v})\end{align*}

**Optimizer.** We use Adam optimizer[45], with $\beta _1 = 0.9, \beta _2 = 0.999$, learning rate = $10^{-3}$, weight decay = $10^{-5}$.

**Pretraining.** To enhance performance, the subgraph encoder will undergo pre-training using graph contrastive learning prior to training. We employ the graph contrastive learning framework as previously introduced in the work of [46]. In our implementation, unlike conventional graphs, the augmentation applied to the link subgraph does not impact the central nodes. Specifically, for the subgraph $G_{u,v}^h$, node dropping during data augmentation does not remove nodes u and v, and edge perturbation during data augmentation does not introduce or remove edges $e_{u,v}$.

### Baseline method

The scHiCluster algorithm, as described in [20], integrates convolution and random walk with restart techniques. The implementation used for scHiCluster can be found at https://github.com/zhoujt1994/scHiCluster, where the default parameters were employed. Higashi, described in [21, 22], utilizes a hypergraph learning model. The implementation used for Higashi can be found at https://github.com/ma-compbio/Higashi, and the default parameters were utilized. scDEC-Hi-C, as proposed in [23], introduces a convolutional generative neural network model. The implementation for scDEC-Hi-C is available at https://github.com/ kimmo1019/ scDEC-Hi-C., and the default parameters were utilized.

### Evaluation metrics for imputation and clustering

In the imputation task, the performance of imputation is evaluated using the Area Under the Precision-Recall Curve and the Area Under the Receiver Operating Characteristic Curve metrics. For clustering tasks, different evaluation metrics are employed based on the datasets used. In the case of the dataset by Ramani et al. [14] and the 4DN-sci-Hi-C dataset [41] with discrete cell states, K-means clustering is applied. The predicted labels are then compared with the true cell types using the ARI metric. For the dataset by Nagano et al. [12] with continuous cell states, the Average Circular ROC metric [42] is used for evaluation.

**Key Points**

- We present HiC-SGL, a novel end-to-end model for imputing scHi-C data based on subgraph extraction and graph representation learning.
- HiC-SGL automatically computes heuristic information for link prediction by extracting k-hop neighborhood subgraphs of the links and feeding them into a subgraph encoder. We compute embeddings that represent the global information of the cell to capture differences in the density, type and cycle state.
- We apply the Transformer architecture to graph neural networks in order to implement the subgraph encoder. Experimental results demonstrate that our model enhances expressive power and achieves better performance by employing a fully connected graph strategy.

## Supplementary Material

supplementary_data_bbad379

## Data Availability

The following publicly available datasets were utilized in our study: ScHi-C datasets of four cell lines obtained from Ramani et al. [14] (GEO: GSE84920). ScHi-C dataset of mouse embryonic stem cells provided by Nagano et al. [12] (GEO: GSE94489). ScHi-C datasets of five cell lines 4DN sci-Hi-C [41] (4dn Data portal: 4DNFIY7AMPUJ, 4DNESUE2NSGS, 4DNESIKGI39T, 4DNES1BK1RMQ and 4DNESTVIP977).

## CODE AVAILABILITY

HiC-SGL is an open-source software based on the pytorch library [47], which can be downloaded from https://github.com/ zhengjh39/HiC-SGL.

## Author Biographies

**Jiahao Zheng** is a student of School of Computer Science and Engineering, Sun Yat-Sen University. His research interests include bioinformatics and machine learning.

**Yuedong Yang** is a Professor of School of Computer Science and Engineering, Sun Yat-Sen University. His research interests include bioinformatics and machine learning.

**Zhiming Dai** is a Associate Professor of School of Computer Science and Engineering, Sun Yat-Sen University. His research interests include bioinformatics and machine learning.
