# Supplementary material for: Subgraph extraction and graph representation learning for single cell Hi-C imputation and clustering
Source: Brief Bioinform. 2023 Dec 1;25(1):bbad379. doi: 10.1093/bib/bbad379 (PMC10691963; doi:10.1093/bib/bbad379)
Supplement: supplementary_data_bbad379 [file supplementary_data_bbad379.pdf]

Supplementary Table 1: Imputation performance(AUPR) by different implements of subgraph encoder.(The first three rows of the table represent the performance of the subgraph encoder in HiC-SGL on three data sets after (1) replacing it with GCN (2) replacing it with GAT (3) removing the attention bias)

	Ramani et al.	Nagano et al.	4DN sci-Hi-C
GCN-encoder	0.737	0.839	0.721
GAT-encoder	0.741	0.852	0.736
Hi-C-SGL w/o atn-bias	0.710	0.771	0.731
**HIC-SGL**	**0.747**	**0.859**	**0.742**

Supplementary Table 2: Imputation performance(AUPR) in different subgraph sampling hop-num(k) on three datasets.(On the Nagano dataset, because the degree of the vertices is large, when k⩾3, the size of the subgraph is close to the entire graph, the computational complexity is too high to obtain results.)

	Ramani et al.	Nagano et al.	4DN sci-Hi-C
k = 1	**0.747**	**0.859**	0.740
k = 2	0.747	0.858	**0.742**
k = 3	0.743	/	0.740

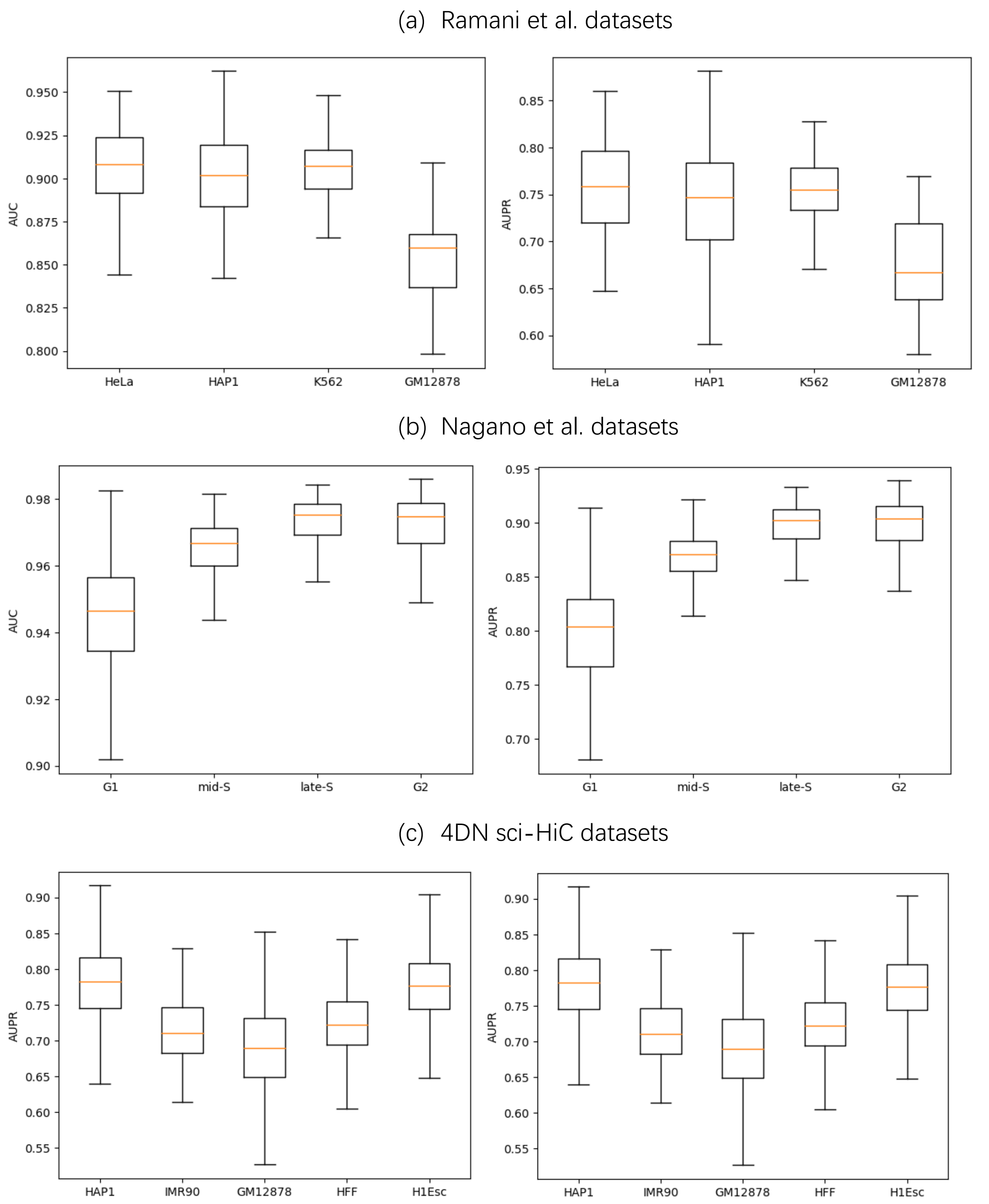

Supplementary Figure 1: Imputation results(left : AUC, right : AUPR) for different cell types on three datasets: (a) Ramani et al. datasets(Hela, HAP1, K562, GM12878); (b) Nanago et al. datasets(G1, mid-S, late-S, G2); (c) 4DN sci-HiC dataset(HAP1, IMR90, GM12878, HFF, H1Esc).

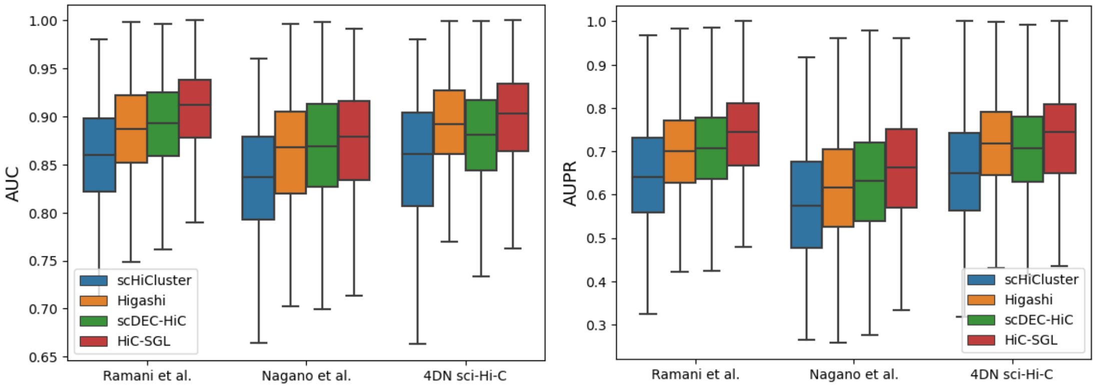

Supplementary Figure 2: Imputation results(left : AUC, right : AUPR) on three data sets with the same resolution (1M), read pairs (2000) and cell number (500)

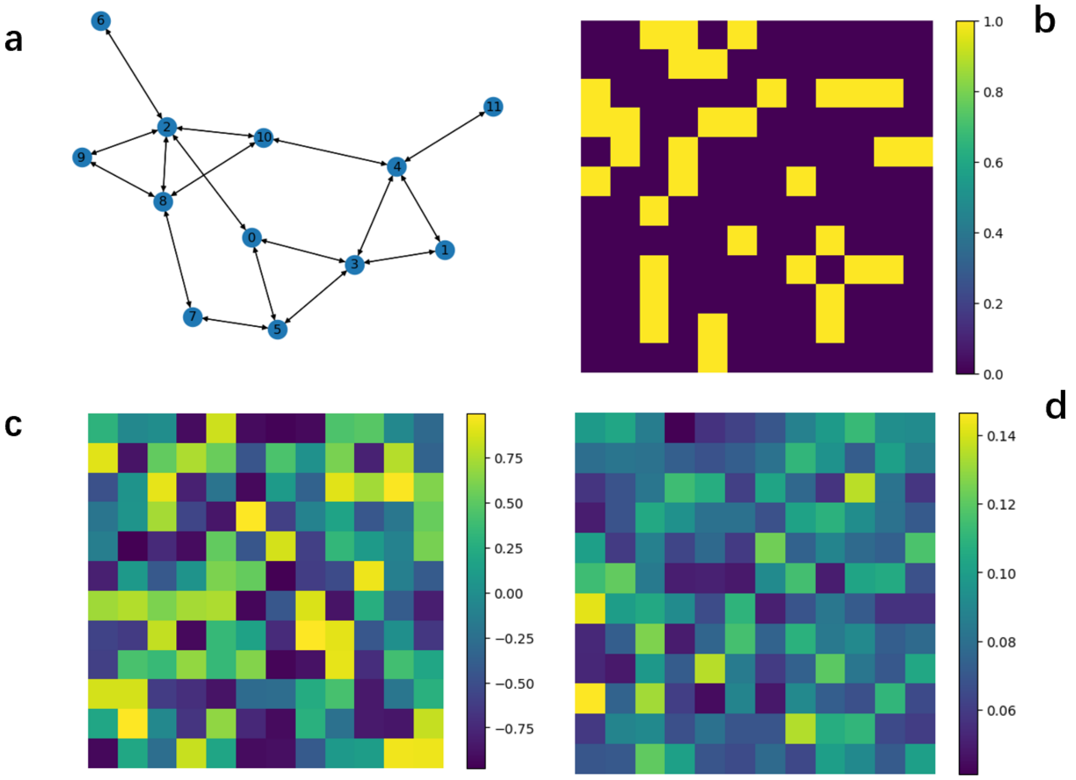

Supplementary Figure 3: (a). 2-hop subgraph with center nodes: (0, 1). (b) adjacency matrix of subgraph. (c) Attention bias calculated from the adjacency matrix by using the trained Hi-C SGL model (d) Attention values on layer 1, head 1.

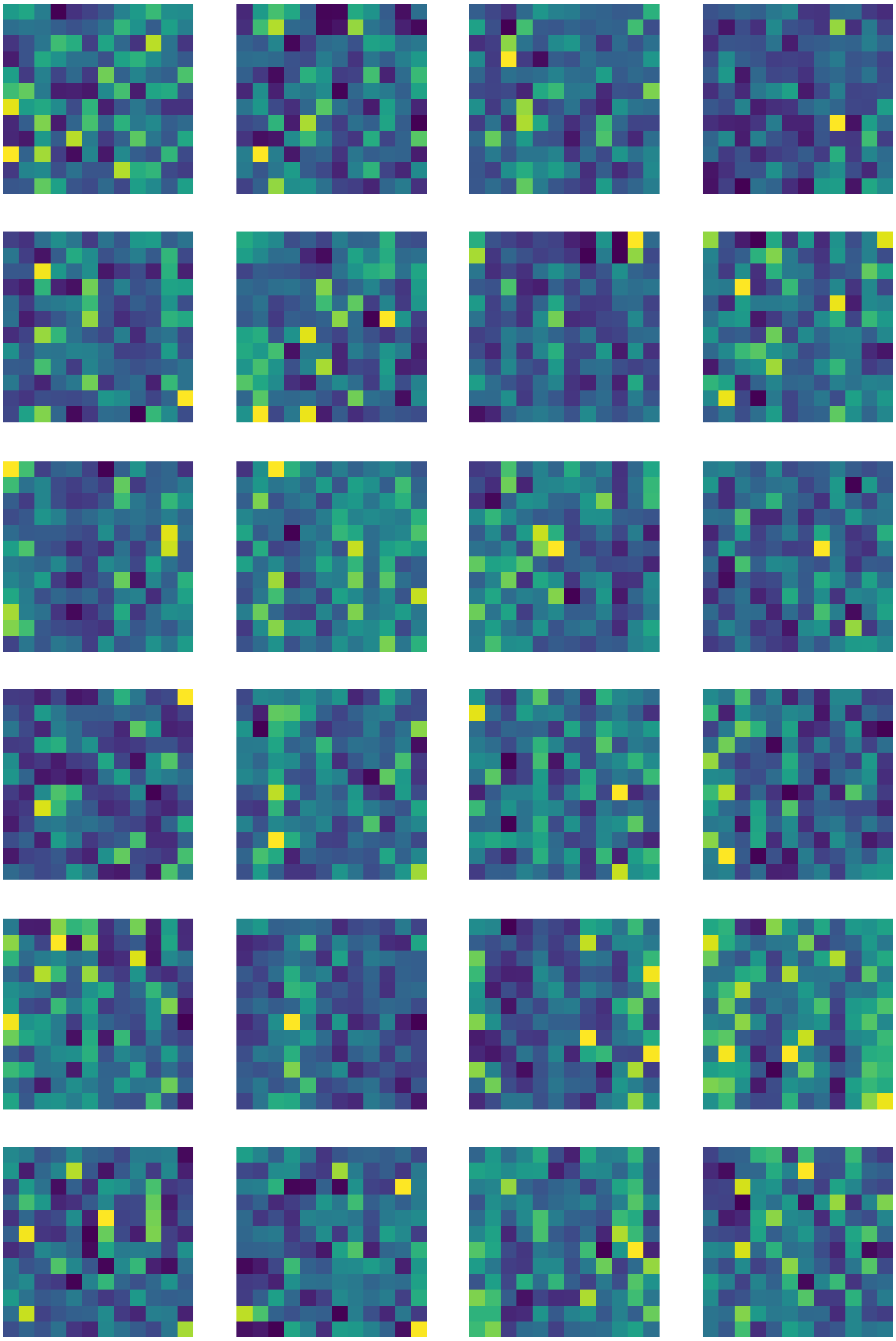

Supplementary Figure 4: Attention values 6 Layers × 4 Heads from subgraph(Fig 1a)
